# Harnessing Nanoporous Hexagonal Structures to Control the Coffee Ring Effect and Enhance Particle Patterning

**DOI:** 10.3390/molecules30153146

**Published:** 2025-07-27

**Authors:** Yu Ju Han, Myung Seo Kim, Seong Min Yoon, Seo Na Yoon, Woo Young Kim, Seok Kim, Young Tae Cho

**Affiliations:** 1 Department of Smart Manufacturing Engineering, Changwon National University, Changwon 51140, Republic of Korea; gksdbwn2164@gs.cwnu.ac.kr (Y.J.H.); 20215000@gs.cwnu.ac.kr (M.S.K.); 20247165@gs.cwnu.ac.kr (S.M.Y.); 20215157@gs.cwnu.ac.kr (S.N.Y.); 2 Global Institute for Advanced Nanoscience & Technology (GIANT), Changwon National University, Changwon 51140, Republic of Korea; wooyoung0329@changwon.ac.kr; 3 Department of Mechanical Engineering, Yonsei University, 50 Yonsei-ro, Seodaemun-gu, Seoul 03722, Republic of Korea; seokkim@yonsei.ac.kr

**Keywords:** coffee-ring effect, nano porous, microstructured, particle aggregation, evaporation behavior, UV-nano imprint lithography (UV-NIL), surface wettability

## Abstract

The coffee-ring effect, while harnessed in diverse fields such as biosensing and printing, poses challenges for achieving uniform particle deposition. Controlling this phenomenon is thus essential for precision patterning. This study proposes a novel method to regulate coffee-ring formation by tuning surface wettability via integrated nanoporous and hexagonal microstructures. Four distinct surface types were fabricated using UV nanoimprint lithography: planar, porous planar, hexagonal wall, and porous hexagonal wall. The evaporation behavior of colloidal droplets and subsequent particle aggregation were analyzed through contact angle measurements and confocal microscopy. Results demonstrated that nanoscale porosity significantly increased surface wettability and accelerated evaporation, while the hexagonal pattern enhanced droplet stability and suppressed contact line movement. The porous hexagonal surface, in particular, enabled the formation of connected dual-ring patterns with higher particle accumulation near the contact edge. This synergistic design facilitated both stable evaporation and improved localization of particles. The findings provide a quantitative basis for applying patterned porous surfaces in evaporation-driven platforms, with implications for enhanced sensitivity and reproducibility in surface-enhanced Raman scattering (SERS) and other biosensing applications.

## 1. Introduction

The surface serves as the initial point of contact where interactions with external substances occur, and by controlling its properties, the interaction between the surface and droplets can be precisely regulated. In particular, the evaporation of colloidal droplets fixed on a solid substrate is a naturally occurring phenomenon frequently observed in everyday life. This simple evaporation process enables effective fabrication techniques that allow precise particle arrangement or coating on surfaces without causing physical damage [1]. Such control has attracted considerable attention for its potential applications in various advanced technological fields, including inkjet printing [2,3], solar panels [4,5], and biosensors [6,7].

The evaporation of colloidal droplets leads to the formation of diverse particle aggregation structures due to the complex interactions among the liquid, vapor, and solid phases [8,9]. A representative example is the coffee-ring pattern, which forms when the contact line remains pinned during droplet evaporation. The difference in evaporation rates between the center and the edge induces Marangoni flow [10], driving suspended particles toward the edge [11,12]. Such coffee-ring patterns exhibit a characteristic tendency to concentrate particles at the edge and are being utilized in various applications, including sensing platforms [13].

Various approaches have been proposed to control the coffee-ring effect, including modifications of the chemical properties of the substrate [14,15], manipulation of the particle characteristics within colloidal droplets [16,17], and regulation of surface wettability [18,19]. Among these, wettability-based control has attracted significant attention due to its simplicity and efficiency, which can be achieved through structural modifications or chemical treatments of the surface. In particular, recent studies have actively explored the use of patterned surface structures [20,21,22] and porous surface properties [23] to modulate wettability. Porous surfaces, in particular, are regarded as an effective means of enhancing surface roughness, thereby enabling precise control over wettability.

However, studies that simultaneously apply both patterned and porous surfaces have rarely been conducted, although their combination is expected to produce synergistic effects in wettability control. Patterned surfaces contribute to the stabilization of the droplet contact line [24] and regulate particle transport, whereas porous structures increase the hydrophilicity of both the substrate and the suspended particles, thereby enhancing overall wettability. This enhanced wettability strengthens contact line pinning and intensifies the outward capillary flow toward the droplet edge, resulting in a more pronounced coffee-ring pattern. Furthermore, modulation of the evaporation rate contributes to improved uniformity in particle aggregation [25].

**Figure 1:**
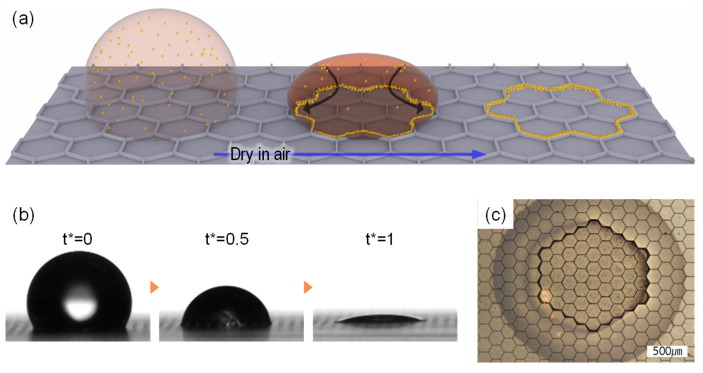
(**a**) Schematic diagram of evaporation behavior and particle aggregation mechanism of a colloidal droplet on a porous micro-hexagonal pattern; (**b**) side view image of the evaporation behavior of a colloidal droplet—time t was normalized to the total evaporation time t*, such that t* = 0 indicates the initial deposition and t* = 1 represents complete evaporation; (**c**) confocal image and SEM image of the aggregation particles.

Therefore, as shown in Figure 1, porous patterned surfaces that integrate these two characteristics provide a novel approach for the precise control of surface wettability and the manipulation of coffee-ring formation, as schematically illustrated by evaporation through the evaporation behavior, contact line dynamics, and final particle deposition patterns.

Figure 1 provides a conceptual overview of the study. Figure 1a illustrates the schematic mechanism of particle aggregation at the droplet edge during evaporation, showing how initially well-dispersed particles on the surface accumulate into a coffee-ring pattern. Figure 1b presents side-view images of the evaporation process, normalized by the dimensionless time t*, where t* = 0 corresponds to the initial moment the droplet is deposited on the surface, t* = 0.5 represents the midpoint of evaporation, and t* = 1 indicates complete evaporation. Figure 1c shows an optical microscope image of the particle distribution after evaporation, confirming the formation of the coffee-ring pattern, through which the effect of the coffee ring can be confirmed.

In this study, we propose a novel approach to precisely control the morphology and localization of particle aggregation by modulating surface wettability through adjustments in substrate surface structure, using nanoscale porosity and three-dimensional (3D) microstructures as key variables. The incorporation of 3D structures contributes to the stabilization of evaporation dynamics and enhances signal reproducibility by increasing the effective surface area. Additionally, the application of nanoscale porous structures enables uniform control over the coffee-ring pattern. By manipulating the aggregation pattern of particles through this method, analytical particles can be selectively concentrated in designated regions. This strategy is expected to improve both the predictability of the active sensing area and the sensitivity of signals in future surface-enhanced Raman scattering (SERS)-based detection platforms [26,27,28].

## 2. Results and Discussion

### 2.1. Droplet Evaporation Behavior on Various Surface Types

**Figure 2:**
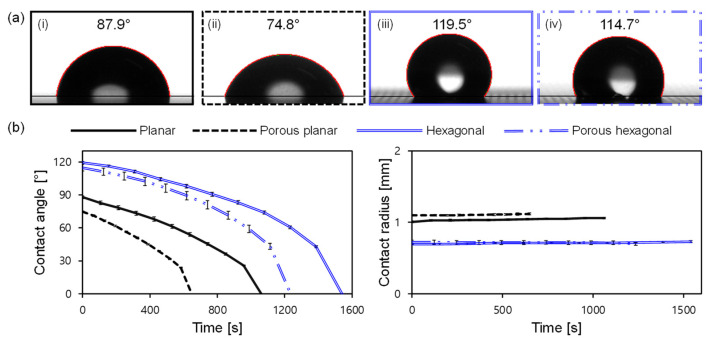
Evaporation behavior of droplets on different surfaces. (**a**) Side view and initial contact angle of sessile drops on different surfaces: (**i**) planar surface, (**ii**) porous planar surface, (**iii**) hexagonal micropatterned surface, and (**iv**) porous hexagonal micropatterned surface; (**b**) graphs of contact angle and contact radius over time on different surfaces. The standard error of the graphs is based on 9 repeated experiments.

Figure 2a shows side-view images of the droplets on each surface, with the numerical values indicating the initial contact angles. Figure 2b presents the changes in contact angle and contact radius over time. For all surface types, the experiments were repeated nine times to calculate the average values and standard deviations. On all four surfaces, the droplets evaporated in the constant contact radius (CCR) mode, in which the contact line remained pinned while the contact angle decreased. Based on these results, the effects of surface patterning and the presence of nanopores on evaporation behavior were analyzed.

The initial contact angle on the hexagonal wall surface was measured to be 119.5°, whereas that on the planar surface was 87.9°, indicating that the hexagonal wall structure contributes to an increase in the initial contact angle. When a colloidal droplet contacts the hexagonal wall surface, the microstructures on the surface generate an air pocket effect, which disrupts direct interaction between the droplet and the solid surface, thereby reducing the solid–liquid interfacial energy. Additionally, the sharp edge effect [29] reduces the actual contact area between the droplet and the surface. As a result, the droplet maintains a higher contact angle on the structured surface [24].

This phenomenon can be interpreted based on the Cassie–Baxter state. The Cassie–Baxter equation describes the apparent contact angle of a droplet on a rough surface when the liquid contacts a composite interface composed of both solid and trapped air [30]. According to Equation (1), the formation of an air pocket is indicated when the solid–liquid contact area fraction (*f_s_*) is less than 1 (*f_s_* < 1).

(1) $$\cos\theta^{*}=f_{s}\left(\cos\theta +1\right)-1$$

In this equation, *f_s_* represents the fraction of the solid surface in contact with the liquid (0 ≤ *f_s_* ≤ 1), θ* is the apparent contact angle on the hexagonal wall surface, and *θ* is the intrinsic contact angle on the planar surface.

By substituting the measured contact angles on the hexagonal wall surface (119.5°) and the planar surface (87.9°) into Equation (1), the solid–liquid contact area fraction (*f_s_*) was calculated to be 0.49. This result quantitatively confirms that the air trapped in the cavities of the hexagonal wall structure induces a hydrophobic surface state, thereby contributing to the increase in contact angle [31,32]. An increased contact angle enables the droplet to maintain a more stable, rounded shape on the surface, minimizing the movement of the contact line and promoting uniform evaporation. Consequently, the droplet maintains a stable morphology during evaporation, allowing for the formation of a more uniform deposition pattern.

On the planar surfaces, the initial contact angle was measured to be 87.9° for the non-porous surface and 74.8° for the porous surface. This indicates that the pore structure of the surface influences the initial contact angle. Such results can be interpreted using the capillary pressure equation and Wenzel’s model.

First, the capillary pressure equation (Equation (2)) shows that the capillary pressure (P) increases as the pore radius (*r_p_*) decreases [33].

(2) $$P=\frac{2\gamma \cos\theta }{r_{p}}$$

In this equation, *γ* represents the surface tension of the liquid, *θ* is the contact angle, and *r_p_* is the pore radius.

On the non-porous planar surface, the pore radius (*r_p_*) is effectively very large, resulting in negligible capillary pressure. In contrast, the formation of nanoscale pores on the porous surface reduces *r_p_* to a finite value, thereby increasing the capillary pressure (P). This increase in capillary pressure enhances the wettability of the porous surface, leading to a reduction in the initial contact angle. In addition, according to Wenzel’s equation (Equation (3)), an increase in the surface roughness factor (*r* > 1) leads to a decrease in the apparent contact angle (θ*), indicating enhanced wettability [34].

(3) $$\cos\theta^{*}=r\cos\theta$$

In this equation, θ* is the apparent contact angle on the porous planar surface, *θ* is the intrinsic contact angle on the non-porous planar surface, and r is the surface roughness factor (*r* > 1).

By substituting the measured contact angles on the porous planar surface (θ*= 74.8°) and the non-porous planar surface (*θ* = 87.9°) into Wenzel’s equation, it was confirmed that the roughness factor *r* satisfies *r* > 1. This indicates that the porous planar surface is in the Wenzel state and that the formation of nanoscale pores increases the surface roughness, thereby decreasing the contact angle and enhancing the surface wettability [35,36].

A decrease in contact angle was found to correlate with an increase in contact radius, which expands the liquid–air interfacial area and thereby promotes faster evaporation. In particular, the presence of nanoscale pores contributes to this enhancement by increasing the effective surface area. However, nanoscale porosity can also lead to asymmetric evaporation, activate contact line movement, and consequently reduce the stability of the evaporation behavior. These characteristics may hinder the formation of uniform deposition patterns.

The initial contact angle on the hexagonal wall structure was measured to be 119.5° for the non-porous surface and 114.7° for the porous surface. Similar to the planar surface case, this result suggests that the formation of nanoscale pores enhances surface wettability. According to previous studies, the capillary pressure equation becomes invalid when the pore radius (*r_p_*) exceeds a certain critical threshold [37,38]. Since the characteristic radius of the hexagonal wall structure (100 μm) exceeds this threshold, only the r_p_ values corresponding to the nanoscale pores were considered in the capillary pressure analysis. In the hexagonal wall structure, the formation of nanoscale pores also led to a decrease in the pore radius (*r_p_*), thereby increasing the capillary pressure and enhancing wettability compared to the non-porous surface. However, in this structured geometry, the wall features act as physical barriers that restrict droplet movement, limiting the effect of capillary forces compared to that observed on the planar surface.

On the planar surface, the decrease in contact angle and the corresponding increase in contact radius due to porosity were more pronounced, suggesting the potential for enhanced control over the evaporation rate. However, this also implies that the evaporation behavior may become more sensitive to variations in pore structure, making precise control more challenging. In contrast, the porous hexagonal wall surface combines nanoscale pores with a hexagonal pattern, offering both reduced evaporation time and stable droplet evaporation behavior [24]. The nanoscale pores enhance surface wettability, thereby accelerating the evaporation rate, while the hexagonal pattern promotes droplet stability by generating air pockets beneath the droplet. As a result, contact line movement is minimized during evaporation, enabling the formation of uniform deposition patterns.

In conclusion, the porous hexagonal wall surface represents an optimized structure that effectively balances evaporation speed and stability through the combined effects of surface patterning and nanoporosity.

### 2.2. Particle Aggregation on Different Surface Types

**Figure 3:**
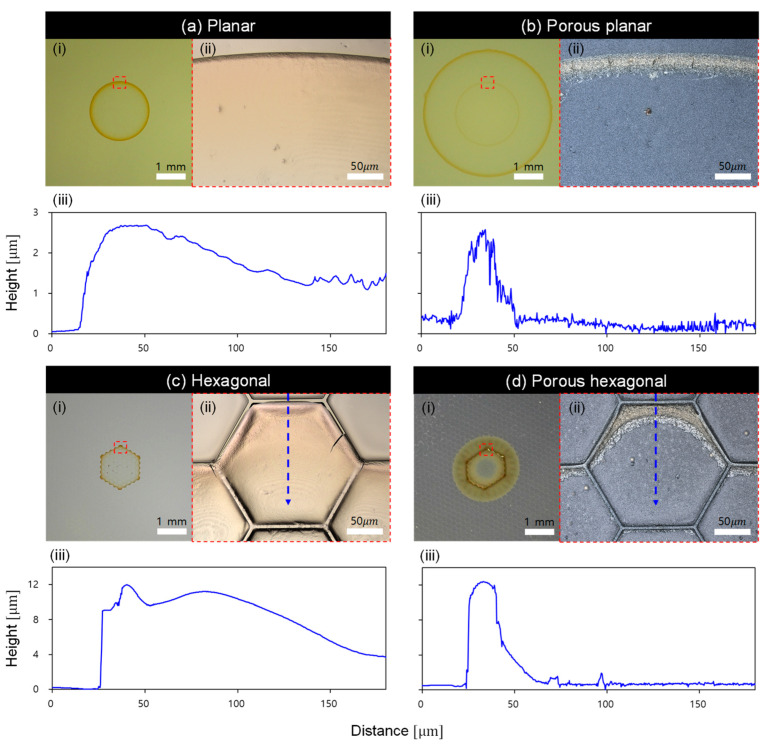
Particle aggregation on four surface types: (**a**) planar, (**b**) porous planar, (**c**) hexagonal, and (**d**) porous hexagonal. For each surface, the following are shown: (**i**) USB microscope overview of the entire coffee-ring deposit (scale bar = 1 mm); (**ii**) optical microscope close-up of the region marked by the dashed red box in (**i**) (scale bar = 50 µm); and (**iii**) height profile of the particle layer measured along the dashed blue line in (**ii**).

Figure 3 illustrates the post-evaporation particle aggregation on each surface. Figure 3a shows images of the aggregated particles captured using a USB microscope, while Figure 3b presents magnified images of the red boxed regions in (a) acquired using an optical microscope. The USB microscope was employed to observe the overall morphology of the particle aggregation, whereas the optical microscope was used to analyze the detailed particle distribution and structure. Figure 3c displays the height profiles of the particles measured along the blue dashed lines in (b), enabling quantitative comparison of particle accumulation height and distribution across the different surface types. Since the evaporation process proceeded symmetrically, the analysis was conducted near the vertex regions of each surface.

On the planar surfaces, distinct differences in the coffee-ring patterns were observed depending on porosity. On the non-porous surface, the initial contact line remained pinned during evaporation, leading to particle accumulation near the contact line and the formation of a circular single coffee ring. In contrast, the enhanced wettability of the porous surface resulted in the formation of two concentric circular rings. In general, for colloidal droplets, multiple concentric rings have been reported to form as a result of repeated pinning–depinning (stick–slip) motion of the contact line during evaporation [39,40,41]. Each pinning phase leads to particle deposition at the contact line, while subsequent depinning generates a new ring, resulting in a classic multi-ring coffee pattern.

However, in the case of the porous surface investigated in this study, a different mechanism appears to govern the deposition. Due to the high intrinsic wettability and capillary absorption of the porous substrate, the droplet rapidly spreads beyond the initial contact line immediately after it is dropped onto the surface, resulting in the early formation of an outer ring. Subsequently, the residual liquid pinned at the original contact line gradually evaporates, forming an inner ring.

As a result, even in the absence of repeated stick–slip events, two distinct concentric rings are formed. This suggests that the enhanced wettability of the porous surface alters the particle transport dynamics, yielding a coffee-ring pattern that is fundamentally different from that on the non-porous surface.

The coffee-ring patterns on the hexagonal wall surfaces also varied depending on the presence of surface porosity. A single ring was formed on the non-porous hexagonal wall surface, whereas connected double rings were observed on the porous hexagonal wall surface. On the non-porous hexagonal wall surface, the droplet remained stable due to the structural confinement, resulting in the formation of a single ring with a hexagonal shape. In contrast, on the porous hexagonal wall surface, the enhanced wettability caused the droplet to spread further, leading to the formation of two interconnected rings.

Notably, on the porous hexagonal wall surface, the two rings were not separately formed as in the planar surface, but rather appeared as a continuous, connected pattern. This difference can be attributed to the structural influence of the surface geometry. On the planar porous surface, there are no structural barriers to restrict fluid flow, allowing the liquid to spread rapidly during evaporation without significant particle accumulation along its path. In contrast, on the porous hexagonal wall surface, the wall structures physically obstruct the outward flow of liquid, causing particles to accumulate along the flow path. This accumulation process occurs progressively along the direction of liquid movement, ultimately resulting in the formation of two connected rings.

As shown in the graph in Figure 3c and the corresponding image analysis, the particle height on the non-porous surface gradually decreased from the contact line toward the center. In contrast, on the porous surface, a steep decline in particle height was observed near the contact line. This difference is attributed to the enhanced wettability and accelerated evaporation rate of the porous surface, which promotes internal mass transport within the droplet. As the evaporation rate increases, the difference in evaporation flux between the contact line and the droplet center becomes more pronounced, intensifying surface tension gradients. This imbalance induces a strong Marangoni flow from the droplet center toward the contact line. As a result, particles are rapidly transported and densely accumulated near the contact line.

## 3. Materials and Methods

### 3.1. Fabrication of Surface Samples

**Figure 4:**
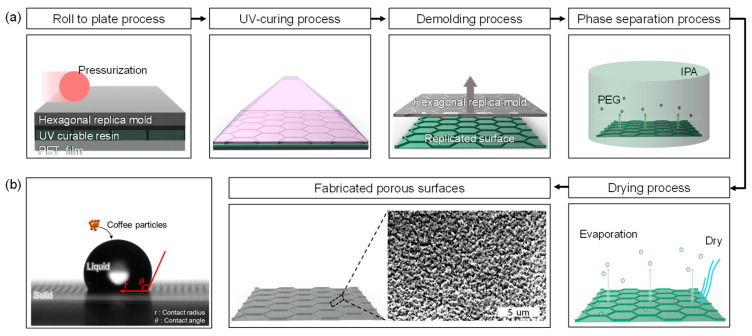
(**a**) Schematics of the porous surface manufacturing process using UV-NIL. PEG was added as a porogen and removed after UV curing, leaving pores in the PUA matrix; (**b**) schematics of a sessile-droplet contact angle and contact radius system.

In this study, various surface geometries were fabricated and analyzed to investigate the influence of surface structures on coffee ring formation. As illustrated in Figure 4a, the surfaces were replicated using UV Nano Imprint Lithography (UV-NIL) [42]. Subsequently, as shown in Figure 4b, 2 μL colloidal droplets were deposited on the fabricated surfaces using the sessile drop method. The aggregation tendency during evaporation was repeatedly measured until complete drying of the droplets, enabling the acquisition of quantitative data. The fabricated surfaces consisted of four types: planar, porous planar, hexagonal wall, and porous hexagonal wall structures. The non-porous surfaces (planar and hexagonal walls) were prepared using a UV-curable polyurethane acrylate (PUA) resin. In contrast, the porous surfaces (porous planar and porous hexagonal wall) were fabricated using a resin composed of PUA mixed with non-curable polyethylene glycol (PEG)at a weight ratio of 1:1. Both resin types were cured by exposure to UV light (365 nm wavelength) for 2 s.

The planar surface was fabricated by applying resin between a polyethylene terephthalate (PET) film and a fluorinated ethylene propylene (FEP) release film, followed by pressing. The hexagonal wall surface was produced using the same method, except that a replica mold with an engraved hexagonal wall pattern was used instead of the FEP film. Porous surfaces were fabricated by first performing the UV-NIL process, as shown in Figure 4a. After UV curing, the unpolymerized PEG, which had been added as a sacrificial agent for pore formation, was removed by dissolving it in isopropyl alcohol (IPA). The surfaces were then dried using an air gun to eliminate any residual solvent, resulting in the formation of a nanoporous surface.

To analyze the structural characteristics of the fabricated hexagonal wall and porous surfaces, surface morphologies were observed using optical microscopy and scanning electron microscopy (SEM). Additionally, pore size distributions were quantitatively evaluated through image analysis.

**Figure 5:**
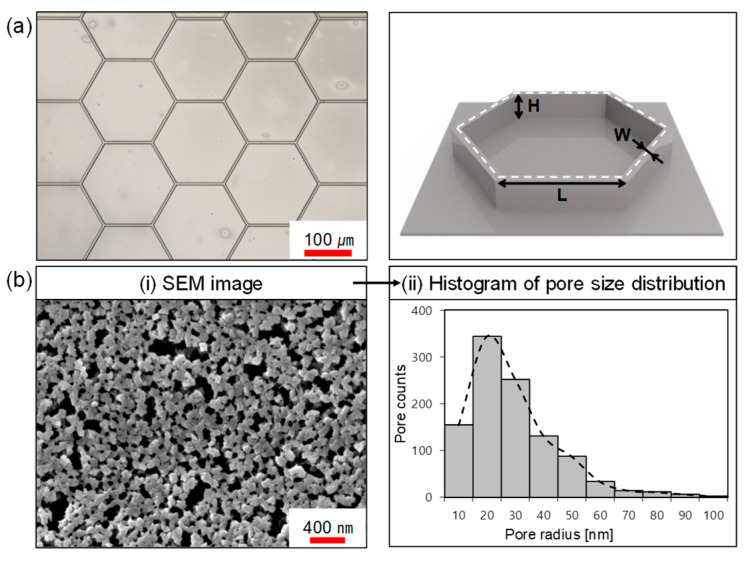
Characterization of the hexagonal and porous surface. (**a**) Hexagonal optical microscope image and pattern size; (**b**) (**i**) pore SEM image and (**ii**) histogram of pore size distribution.

Figure 5a presents an optical microscope image of the fabricated hexagonal wall surface, in which the side length of the hexagon is 100 μm, the wall thickness is 3 μm, and the wall height is 10 μm. Figure 5b shows the characterization of the porous surface: (i) an SEM image of the porous surface, (ii) a total of 1036 pores measured from three different SEM images of the porous surface, and a histogram representing the pore size distribution was generated. The original SEM images and their corresponding binarized versions are provided in Figure S1.

In the case of porous surfaces, it is challenging to clearly define the boundaries between pore and solid regions in SEM images, as the pores are irregular in size and exhibit interconnected morphologies. Due to these complexities, simple dimensional measurements are insufficient for reliable quantitative analysis. To address this issue, the SEM images were binarized to isolate the pore regions, and quantitative evaluation of the pore size distribution was performed through histogram analysis [43,44]. This approach allows not only the visualization of the average pore size but also the distribution range, enabling a more effective assessment of the uniformity and reproducibility of the porous structure.

The SEM images were binarized using a threshold value of 0.3 to extract the pore regions, and the number of pixels within the extracted areas was calculated to determine the pore area. The pixel-based measurements were then converted into actual area values in nanometers (nm^2^) using the scale bar provided in the SEM images. Based on this analysis, the pore size distribution was evaluated to quantitatively characterize the porous surfaces. The measurement results showed that the pore sizes ranged from 10 to 100 nm, with pores around 20 nm in size exhibiting the highest frequency. Notably, pores smaller than 50 nm accounted for 94% of the total, indicating that the porous surfaces used in the experiments were predominantly composed of uniformly distributed sub-50 nm nanopores.

### 3.2. Evaporation and Particle Aggregation Methods

Coffee particles, which are easily accessible and readily miscible with water, were selected as the colloidal material. A colloidal solution was prepared by mixing coffee particles (Kanu, Dongsuh Foods, Incheon, Republic of Korea) with water at a concentration of 0.31 wt%, followed by stirring at 600 RPM for 10 min using an overhead stirrer.

The prepared colloidal solution was precisely dispensed onto the surfaces in 2 μL volumes using a contact angle meter (SmartDrop, Femtobiomed Inc., Seongnam, Republic of Korea) to analyze the evaporation behavior. Additional measurements were also performed using higher concentrations (1.5 wt% and 3 wt%) to examine the effect of particle loading on ring formation (Figure S2). All experiments were conducted in a Class 1000 cleanroom, where the temperature and relative humidity were maintained at 20.8 ± 0.6 °C and 40.8 ± 1.9%, respectively. The contact angle and contact radius were recorded at 1 min intervals using the static contact angle method. To ensure data reliability, each surface type was measured nine times and quantitatively analyzed. In this context, the contact angle refers to the angle formed between the droplet and the solid surface upon contact, while the contact radius represents half the length of the contact line between the droplet and the surface.

After complete evaporation, the deposited particles were imaged using a laser scanning confocal microscope (Keyence, VK-X1000, Seoul, Republic of Korea) and a USB microscope (Dino-Lite, AM-4113T, Seoul, Republic of Korea). The height profile of the particles near the contact line was analyzed to investigate the distribution and aggregation patterns of the particles according to surface geometry.

## 4. Conclusions

In this study, the effects of surface geometry and nanoscale porosity on evaporation behavior and particle deposition were systematically investigated using four types of UV-NIL-fabricated surfaces. The porous surfaces showed improved wettability, larger contact radii, and faster evaporation, while hexagonal wall structures provided confinement that influenced ring morphology. Notably, the porous hexagonal wall structure enabled the formation of dense, edge-focused particle rings due to restricted spreading and Marangoni-enhanced flow.

All droplets evaporated in the CCR mode, resulting in distinct single or double-ring patterns depending on the surface design. These findings demonstrate that combining nanoscale porosity with 3D microscale structuring offers an effective strategy to control particle aggregation and coffee-ring patterns in evaporation-driven processes.

Although this study used coffee particles as model colloids, the results serve as a foundational step for developing advanced biosensing platforms. Future work will evaluate the sensing performance of these porous structured surfaces using real analytes such as proteins, DNA, or nanoparticles, with a focus on reproducibility and signal enhancement.

## Data Availability

The original contributions presented in this study are included in the article. Further inquiries can be directed to the corresponding author.

## Supplementary Materials

The following supporting information can be downloaded at: https://www.mdpi.com/article/10.3390/molecules30153146/s1, Figure S1: (a) SEM images taken at three different locations on the porous surface; (b) Corresponding binarized images obtained from the SEM images to analyze pore distribution and area fraction. All scale bars represent 400 nm. Figure S2. Colloidal droplets containing coffee particles (concentrations: 0.3 wt%, 1.5 wt%, and 3 wt%) were deposited (2 μL each) onto four different surface types (planar, porous planar, hexagonal pattern, and porous hexagonal pattern), dried completely, and imaged from the top view using a USB microscope. USB microscope overview of the entire coffee-ring deposit (scale bar = 1 mm).
