# Supplementary material for: Harnessing Nanoporous Hexagonal Structures to Control the Coffee Ring Effect and Enhance Particle Patterning
Source: Molecules. 2025 Jul 27;30(15):3146. doi: 10.3390/molecules30153146 (PMC12348240; doi:10.3390/molecules30153146)
Supplement: Supplementary file 1 [file molecules-30-03146-s001.zip › molecules-3729624-supplementary.pdf]

Supporting Information

Harnessing Nanoporous Hexagonal Structures to Control the Coffee Ring Effect and Enhance Particle Patterning

Yu Ju Han [1], Myung Seo Kim [1], Seong Min Yoon [1], Seo Na Yoon [1], Woo Young Kim [2], Seok Kim [3] and Young Tae Cho [1,*]

1 Department of Smart Manufacturing Engineering, Changwon National University, Changwon 51140, Republic of Korea; gksdbwn2164@gs.cwnu.ac.kr (Y.J.H.); 20215000@gs.cwnu.ac.kr (M.S.K.); 20247165@gs.cwnu.ac.kr (S.M.Y.); 20215157@gs.cwnu.ac.kr (S.N.Y.)

2 Global Institute for Advanced Nanoscience & Technology (GIANT), Changwon National University, Changwon 51140, Republic of Korea; wooyoung0329@changwon.ac.kr

3 Department of Mechanical Engineering, Yonsei University, 50 Yonsei-ro, Seodaemun-gu, Seoul 03722, Republic of Korea; seokkim@yonsei.ac.kr

* Correspondence: ytcho@changwon.ac.kr

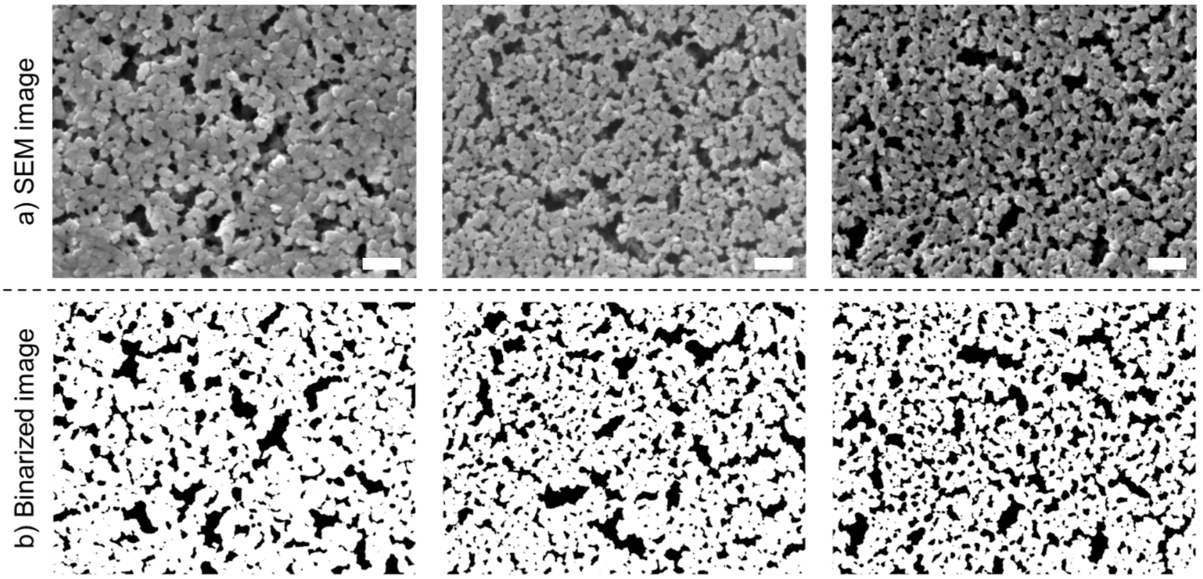

Figure S1. a) SEM images taken at three different locations on the porous surface; b) Corresponding binarized images obtained from the SEM images to analyze pore distribution and area fraction. All scale bars represent 400 nm.

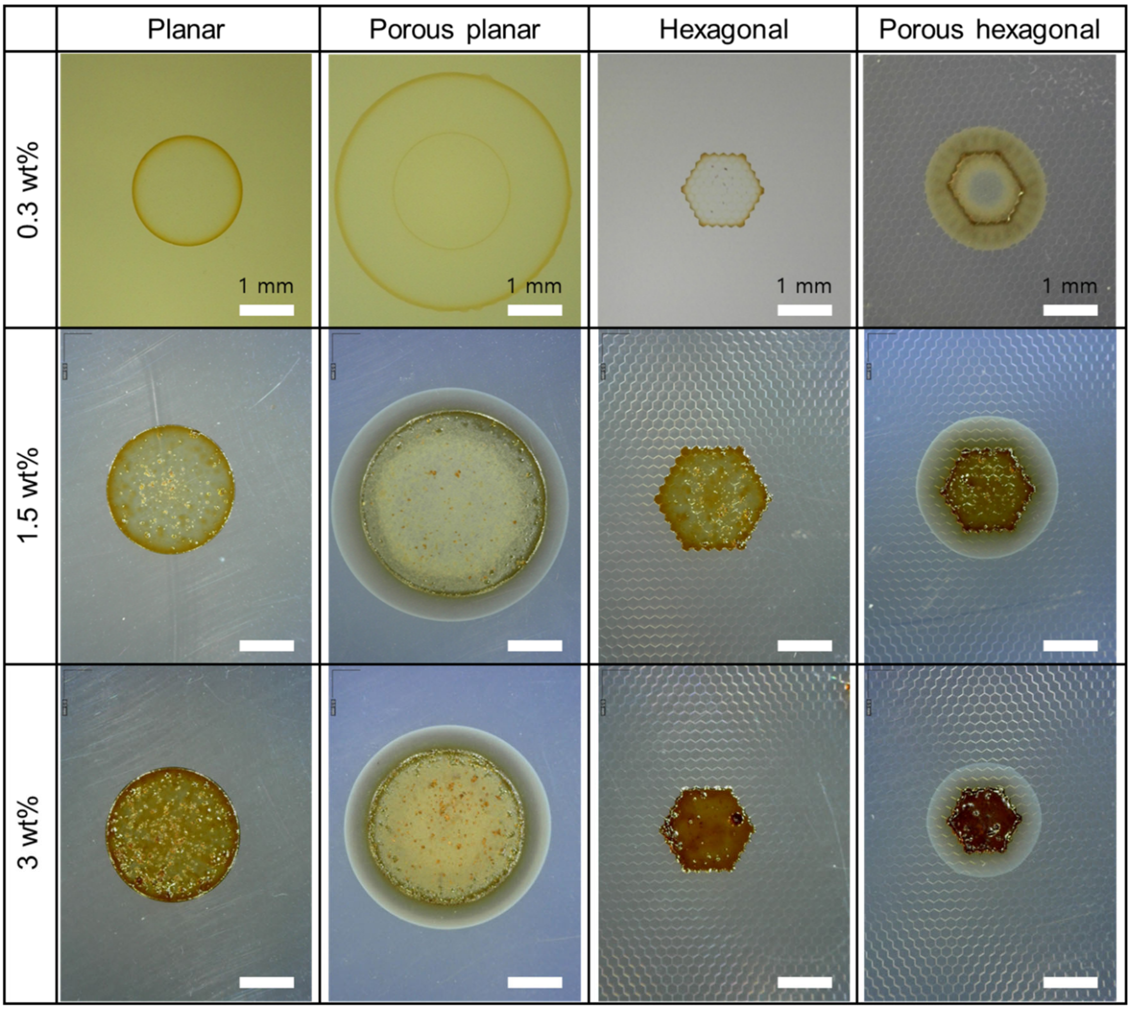

Figure S2. Colloidal droplets containing coffee particles (concentrations: 0.3 wt%, 1.5 wt%, and 3 wt%) were deposited (2 μL each) onto four different surface types (planar, porous planar, hexagonal pattern, and porous hexagonal pattern), dried completely, and imaged from the top view using a USB microscope. USB microscope overview of the entire coffee-ring deposit (scale bar = 1 mm).
